# The effect of kinesiology taping on gait variability in healthy dogs

**DOI:** 10.3389/fvets.2025.1650607

**Published:** 2025-12-08

**Authors:** Ching-Ming Liu, Syu-Lun Lin, Hsiao-Man Liu, Janice L. Huntingford

**Affiliations:** 1 National Taiwan University Veterinary Hospital, Taipei City, Taiwan; 2 Information School, University of Washington, Seattle, WA, United States; 3 Essex Animal Hospital, Essex, ON, Canada

**Keywords:** kinesiology taping, dog, pressure sensitive walkway, kinetic variable, temporospatial variable

## Abstract

**Introduction:**

Kinesiology taping is commonly utilized in sports and rehabilitation for both humans and horses. Evaluating its effectiveness includes both subjective and objective kinesiology assessments. However, there is limited research on the use of kinesiology taping in dogs. This study examines the effects of kinesiology taping on gait variability in healthy dogs.

**Materials and methods:**

A total of eight client-owned dogs varying in age, breed, and body weight were recruited for the study. Three taping methods were applied to the biceps femoris muscle. Mobility, as well as static and dynamic parameters, were assessed using a scale, a stance analyzer, and a pressure-sensitive walkway for data collection and statistical evaluation.

**Results:**

Kinesiology taping on clinically healthy dogs had minimal impact on mobility and stance, and only a partial intrinsic effect on gait. Only 3 of 29 kinetic and temporospatial parameters showed a statistically significant difference (*p* < 0.05) between taping types. Over days with different taping methods, the number of significantly affected kinetic and temporospatial variable items ranged between 2 and 7 out of 29, with inconsistent distribution. The effect of taping on gait also varied depending on whether hair was present or clipped.

**Discussion:**

These findings suggest that kinesiology taping has minimal and inconsistent effects on gait variables in healthy dogs, with limited variation across taping methods and a moderate influence due to the presence of hair. This study provides preliminary data on intrinsic neuromuscular modulation in response to skin stimulation, enhancing our understanding of canine gait biomechanics. The insights gained may help guide future research into dynamic stability, compensatory strategies, and neurosensory responses during healthy movement in healthy dogs.

## 1 Introduction

Kinesiology taping (KT) was developed by a Japanese chiropractor, Kenzo Kase, in the 1970s. This technique involves the use of a pharmaceutical-free elastic woven cotton strip with a heat-sensitive acrylic adhesive, which is applied to the skin. It is designed to treat various musculoskeletal issues in clinical settings and to enhance muscle function in athletes in human medicine (1, 2). Numerous effects of KT have been hypothesized, including pain reduction, wound edema reduction, normalization of muscle function, and improvement of proprioceptive feedback. Various clinical effects of KT have been reviewed in a diversity of conditions and populations (3). Unlike conventional sports tape, which limits movement, the effect of KT is to promote movement. It mimics the thickness and flexibility of the skin and provides support to muscles without restricting the range of motion of limbs.

There are three main functions of KT. First, it decreases local edema by lifting the skin and promoting blood and lymph flow underneath the dermis (4, 5). Second, it stimulates cutaneous mechanoreceptors, which increase afferent feedback to the central nervous system, thus reducing pain sensation (6). Finally, it stimulates the peripheral nerves, lowering the firing threshold of the motor neurons, enhancing the recruitment of motor units, and increasing the excitability of the motor cortex, thereby improving muscle function (7). The recruitment of the muscle spindle fibers via the sensorimotor pathway improves muscle tone (8). Two types of KT techniques are used to produce different effects on muscle function— facilitation and inhibition. Kase (54), who developed the original concept, proposed that applying the tape in the direction of muscle contraction facilitated muscle function, whereas applying it in the opposite direction inhibited it. Tape recoil resembles muscle contraction, moving toward the anchor. When applied in the same direction as the muscle origin (fixed) to the insertion (movable), it enhances contraction. In contrast, applying tape from the insertion to the origin may inhibit contraction. In humans, for example, thigh-to-knee taping boosts quadriceps muscle activity. A neutral application involves no tension and is not intended to affect muscle activity. However, systematic reviews of KT research have shown inconsistent evidence regarding the effectiveness of tape direction in enhancing muscle strength (9).

In humans, KT has been used as an adjunctive therapy for decreasing post-mastectomy lymphedema (10, 11) and for alleviating pain in plantar fasciitis (12), knee osteoarthritis (6), shoulder dysfunction (13), and carpal tunnel syndrome (14). Other adjunct uses are for the treatment of cerebral palsy (8), muscular recovery (15), endurance and motor control performance enhancement for athletes, and the prevention of injuries (9).

KT has been used in the equine athlete for both competition enhancement and rehabilitation (16). It can be used to assess and treat muscular conditions, postural imbalances, and fascia restrictions. The effects on muscles, tendons, and ligament injuries are well addressed, and according to Molle (16), KT can be used in neurologic pathologies as well as lymphatic conditions.

In dogs, KT has been used in lymphatic conditions (17) for the assistance and treatment of muscular conditions, postural imbalances, fascia restrictions, and gait disorders, but there is a paucity of related research. Canine KT books have been published (18, 19), and KT training courses exist; however, there is a lack of evidence about the effect of KT on the gait performance of dogs or any of the other conditions that KT has been proposed to treat in the canine.

Several quantitative gait analysis systems have been validated for the evaluation of gaits in dogs (20). These systems include, but are not limited to, image capture systems, force plates, weight distribution platforms, pressure-sensitive walkways, and inertial measurement units (IMUs) (20, 21). Stance analyzers were utilized to detect static weight distribution on limbs with orthopedic disease (22–24) and to evaluate the outcomes of orthopedic surgery (25, 26). The pressure-sensitive walkway detects both force and non-force information about gait, as well as specific gait patterns for different breeds and sizes of dogs (27–29), dogs with neurological or orthopedic disease (30–32), and rehabilitation outcomes (33).

The purpose of this study was to determine the effects of KT on the gait of clinically healthy dogs, whether different types of KT (facilitation, inhibition or neutral) on biceps femoris muscle would affect the gait, and if the KT effect would be changed by shaving the hair over the muscle. We hypothesized that there would be differences in gait variables with different KT methods on normal dogs and that the hair would affect the effects of taping.

## 2 Materials and methods

### 2.1 Experimental design and data collection

This clinical research was a prospective, randomized, double-blinded, crossover study on the effect of KT in eight client-owned dogs, which were recruited at National Taiwan University Veterinary Hospital. The protocol was reviewed and approved by the National Taiwan University Institutional Animal Care and Use Committee, and the number was NTU-111-EL-00056. The inclusion criteria included healthy client-owned dogs, 1 to 15 years of age, and between 5 kg and 30 kg body weight of any breed, gender, age, and size. Dogs were excluded if they had abnormal mobility, were allergic to the taping, or were difficult to handle for the stance or gait evaluation. The dog owners were informed and signed the consent form before enrolling their animals in the study. After normal physical, orthopedic, and neurological examinations, the dogs were enrolled in the trial. The trial consisted of a baseline period folowed by three consecutive randomized taping periods: K + (facilitation), K− (inhibition), and K = (neutral or no effect). Washout periods were included between each taping period.

The first 2 days of baseline data collection were without taping after clipping the hair on the left thigh using a 2-mm clipper (C6-PetPro, ELEMENT, Huei You Trading Co., New Taipei City, Taiwan). For the first 2 days, the disability score Cincinnati Orthopedic Disability Index (CODI) was evaluated in each dog, and the data for static analysis using a stance analyzer (Companion Stance Analyzer; LiteCure LLC^®^, Newark, DE, USA) were collected. In the CODI questionnaire, there were eight items—walking, running, jumping, getting up, lying down, climbing stairs, descending stairs, and posturing to urinate or defecate—and the owner selected the five situations their dog most frequently experienced. In the stance analysis, dogs were guided by handlers to stand naturally on the weight distribution platform, placing one foot in each quadrant, with a balanced center of gravity and head facing forward. At least 10 readings were taken per dog, and the 5 most consistent values were used. Limb weight distribution was expressed as body weight percentages. Following the static stance analysis, dynamic gait analysis was performed, consisting of five valid passes of walk and five valid passes of trot with a velocity range of ±0.3 m/s and an acceleration of ±0.5 m/s^2^. For this analysis, the dog was loosely leashed by a handler at the left side with their own comfortable speed of walking, and the data were collected from the pressure sensitive walkway (PSW) chipboard and platform equipped with 15,360 sensors covering an area of 203 × 54.2 cm working with a measuring frequency of 100 Hz (CaniGait; FDM Type 2 from Zebris Medical GmbH, Allgäu, Germany). The walkway was covered with a 1.5-mm-thick yoga mat made of natural rubber (TAIMAT, Taiwan) to prevent irritation and slipping.

**Figure 1:**
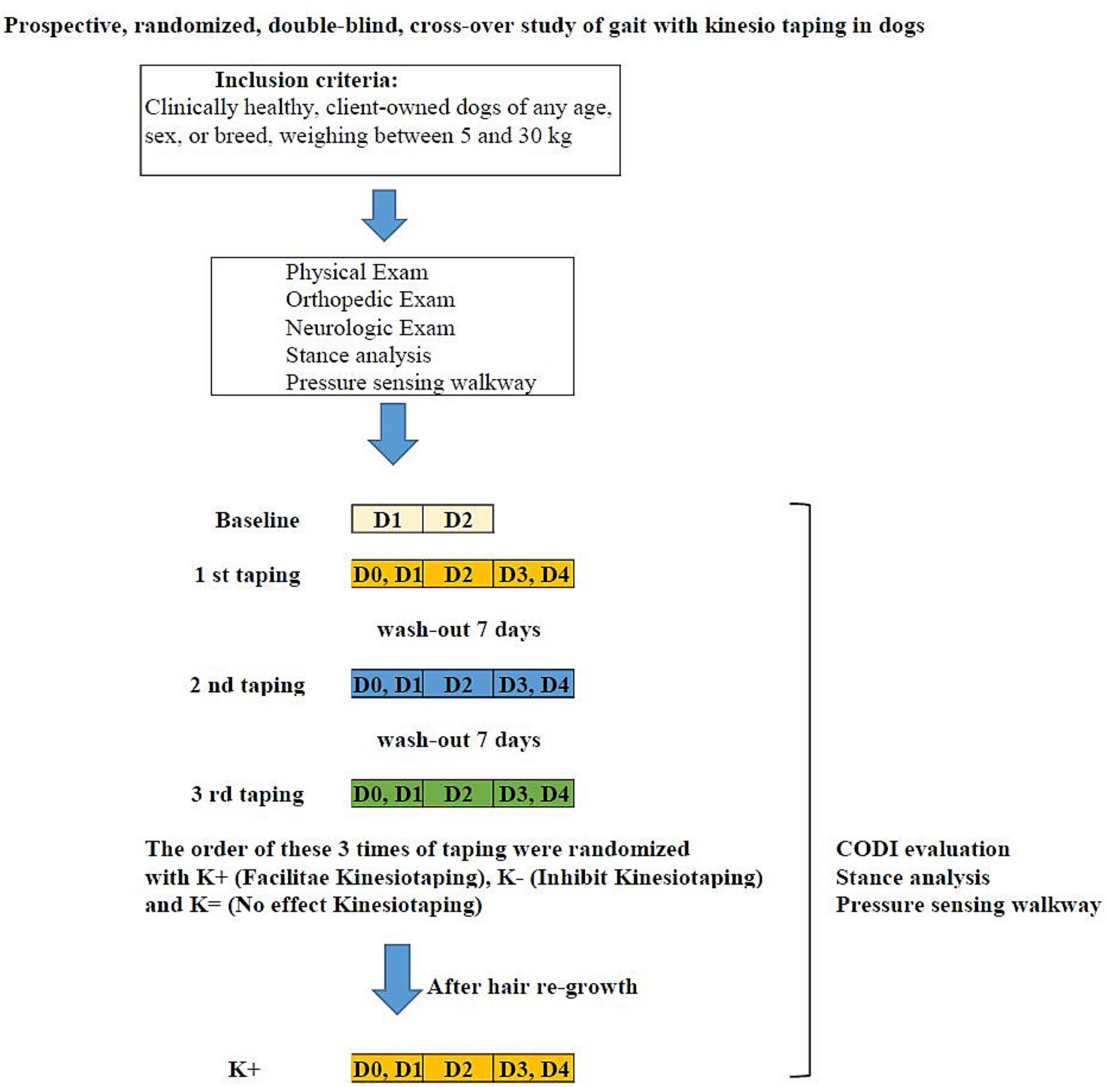
Experimental design.

After the baseline training, the dogs were randomized into three groups by online randomizing software (RANDOM. ORG) as to which type of taping would be received. Every dog eventually received each of the taping groups K+, K−, and K=. After measuring and recording the length needed to cover the interested area, the taping was performed on the left thigh with I-strip 5-cm wide kinesiotape (KINESIO CANINE; Kinesio Holding Corporation, New Mexican, USA) covering the origin and insertion of the biceps femoris muscle. The taping steps followed the standard taping procedure on CANINEEXERCISES website. Kinesio tape contains elastic fibers aligned lengthwise. When the tape is stretched, its recoil creates a force directed toward the end that was first anchored. If this force vector follows the direction of muscle contraction, it facilitates the muscle function, which means helping the muscle contraction; if it goes in the opposite direction, it inhibits it, which means hindering muscle contraction. The spray glue (Adherent; Mueller Sports Medicine, WI, USA) was used on the anchor and the endpoint to make the tape stick firmly at two ends. The direction of K + was from the origin to the insertion of the biceps femoris muscle, that is, from the sacrotuberous ligament to the medial tibial tuberosity. The direction of K− was opposite to K+. Both K + and K− used 50% recoil tension by stretching the tape to extend with 50% available length. The K = taping used 0% recoil tension taping from either end. The tape was left on for 3 days, after which it was removed, and a 7-day washout period with no tape on the dog occurred before it received the next taping. (Figure 1).

The static stance and the gait of walk and trot, as well as the CODI, were measured before taping (D0) and were measured 15 min after taping on Day 1(D1). On the second day (D2), the CODI, the static stance, and the gait of walk and trot were evaluated. On the third day (D3), the CODI and the static stance and the gait of walk and trot were evaluated, and then the tape was removed and the same measurements were retaken (D4). All the measurements were performed in the morning between 9 a.m. to 11 a.m., the same time as D1. Between the trial groups, there was a wash-out period without taping for at least 7 days. After all the dogs completed all three taping groups, the hair was allowed to grow, and then the K + taping was applied using the same method as previously used for the K + taping. The pressure-sensitive walkway recorded two groups of variables of gaits: kinetic gait variables and temporospatial variables. The kinetic gait variables, comprising 10 force-related items, included vertical impulse and average maximal force for all four limbs, as well as bilateral symmetry. The temporospatial variables, consisting of 19 mobility-related items, included velocity (1 item), stride length (1 item), cadence (1 item), step length (4 items), step width (2 items), hind limb reach on both sides, and stance and swing phases for all four limbs.

**Figure 2:**
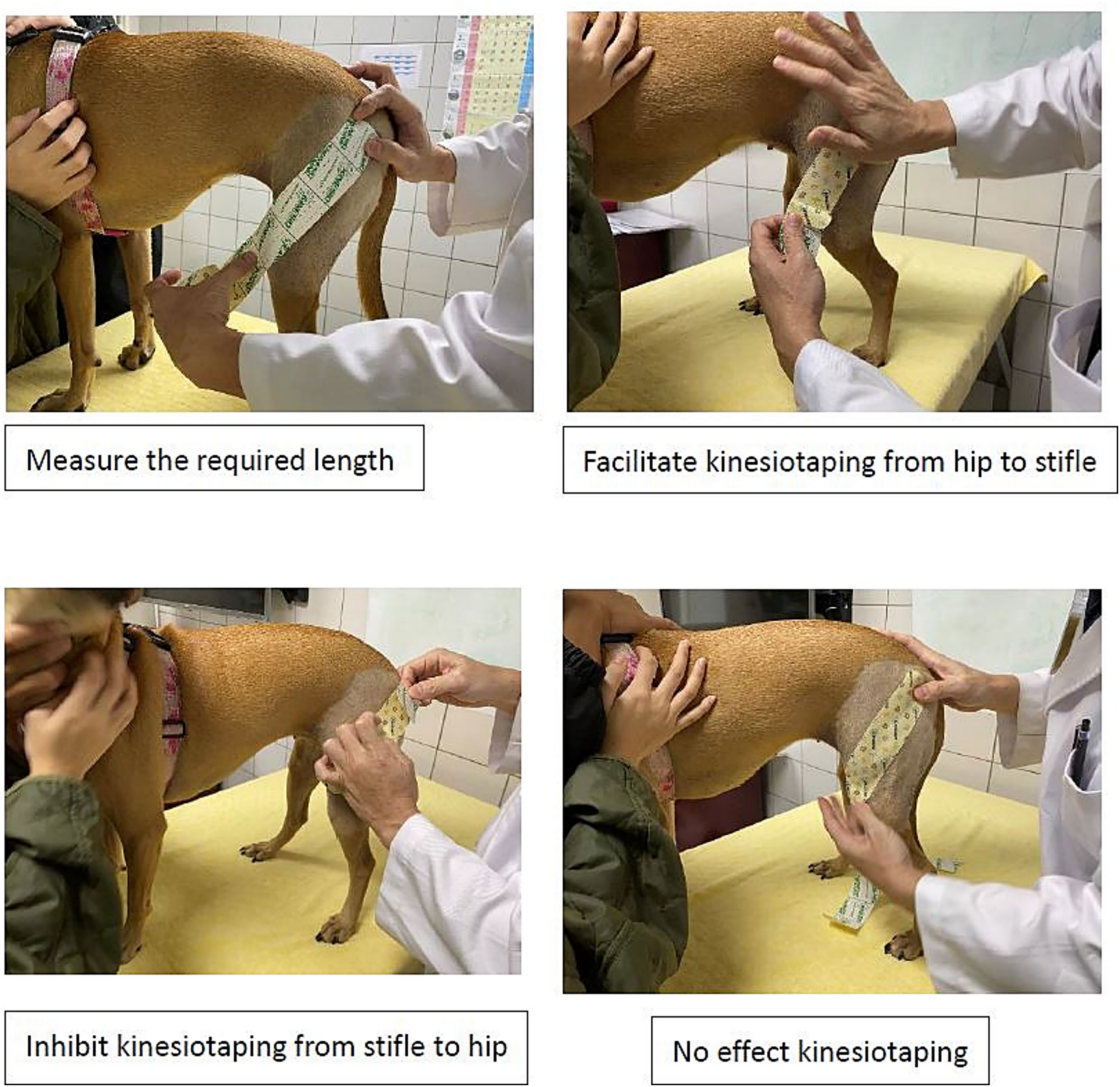
The taping methods.

The taping was performed by a veterinarian (CML) with both KT and canine rehabilitation training (CKTP-certified kinesio taping practitioner and CCRP-certified canine rehabilitation practitioner) (see Figure 2). The evaluator was another veterinarian (SLL) with CCRV (certified canine rehabilitation veterinarian). The randomization was blinded from the owners and the evaluator. All the measured data were collected for statistics.

### 2.2 Statistical analysis

This study contained repeated measurements with designed conditions. Comparative analysis of the three taping methods was conducted over time for all dogs, and gait performance was examined with respect to clipped versus haired taping. For dogs using different taping methods, comparisons were also made on the same day (Day 1, Day 2, and Day 3), with one point assigned per item if a statistically significant difference was found (*p* < 0.05). The data were analyzed by IBM software SPSS version 25. The collected static stance data, dynamic kinetic, and temporospatial data were a number of unpaired samples that originated from the same population, which were non-normally distributed by the Kolmogorow–Smirnov test. They were compared between three or more than three groups with the Kruskal–Wallis test. The collected data were also compared in pairs using the Mann–Whitney test within different groups, Wilcoxon test between the same group under different conditions. For all statistical analyses, values of *p* < 0.05 were considered significant.

## 3 Results

Eight dogs were included in the data analysis, including one Husky, two Labrador Retrievers, two Golden Retrievers, two Mixed breeds, and one Yorkshire Terrier. The mean age was 7.7 years (7.7 ± 4.43 years, range from 1.5–12 years) while the mean body weight was 21.6 kg (21.6 ± 8.62 kg, range from 6.2–30.6 kg). Three dogs were castrated males, four were spayed females, and one dog was an intact female.

The CODI was performed on every visit (total 11 times), and all showed a score of 0. The observed items were scored by clients, and the results of 11 scores, which included baseline two evaluations and three sets of three evaluations in each dog, did not show any changes on baseline, during taping periods, and taping with hair (another three evaluations were after the hair grew).

For the static stance, there were 17 measurements, which were 2 measurements on the baseline and 3 sets of 5 measurements in each dog, including the average stance of the left front limb, the average right front limb, the average left hind limb, and the average right hind limb. The results of the comparison of four stance variables between three different taping methods (*n* = 8) showed only one significant difference (*p* < 0.05) on the right front limb, which was on the diagonal to the taped limb. The *post hoc* analysis showed that the significant difference was between no effect taping and inhibition taping (Table 1).

**Table 1:** The comparison of three different taping methods in stance.

All dogs (*n* = 8) clipped taping in stance			Stance (% of body weight)
			Lt_FL	Rt_FL	Lt_HL	Rt_HL
Facilitate taping period (K+)		Mean	31.14	31.24	18.81	18.87
		SD	6.07	6	3.87	4.96
		Median	32	30	19	19
		CI	19.24−43.04	19.49−43	11.23−26.39	9.14−28.59
No effect taping period (K=)		Mean	31.61	30.38	19.33	18.68
		SD	6.78	6.2	4.16	4.56
		Median	32	30	18.5	18
		CI	18.33−44.89	18.22−42.53	11.18−27.49	9.75−27.62
Inhibit taping period (K−)		Mean	29.83	32.35	19.61	18.22
		SD	7.03	6.03	4.45	5.52
		Median	31	32.5	20	18
		CI	16.04−43.61	20.53−44.17	10.89−28.32	7.41−29.03
K-W test (*p*-value < 0.05)			0.051	0.03	0.467	0.418
K + VS. K=				0.294		
K + VS. K−			0.113		
K = VS. K− (*p*-value < 0.05)				0.009		

Lt_FL, left front limb Rt_FL, right front limb Lt_HL, left hind limb Rt_HL, right hind limb.

SD, Standard deviation; CI, Confidence intervals; K–W test, Kruskal–Wallis test.

There were 17 measurements for the PSW analysis when comparing three different methods of taping for 48 hours at the walk. The comparison of 29 variables showed 3 significant differences (*p* < 0.05), which were on the vertical impulse of the left hind, and the average maximal force of left hind and right hind on the measurements between three different taping methods on the walk (*n* = 8). The value was K = higher than K + , and K + higher than K−. *Post hoc* analysis showed there was no significant difference between K + and K=. When comparing K + with K− and K = with K−, it showed differences (*p* < 0.05) in the vertical impulse of the left hind and the average maximal force of the left hind, which were both on the taping limb. When comparing K = with K−, they showed differences (*p* < 0.05) on the vertical impulse of left hind, and average maximal force of left hind and right hind. There were 3 significant differences (*p* < 0.05), which were on hind step width, left hind reach, and symmetry of front on the measurements between three different taping methods on trot (*n* = 8). In hind step width, the value of K− was higher than K+, and K + was higher than K=. In the left hind reach, K + was higher than K−, and K− was higher than K=. In symmetry of front, the value of K = was higher than K+, and K + was higher than K−. The *post hoc* analysis showed there were differences (*p* < 0.05) in all these three variables when comparing K + with K=. There was no difference in all these three variables when comparing K + with K−. There were differences (*p* < 0.05) in hind step width and symmetry of front when comparing K = with K−. (Table 2; for detailed data, see Supplementary Material).

**Table 2:** The comparison of the measurements with significant differences in walk and trot with three different taping methods (*n* = 8).

Variables		Taping	Mean	SD	Median	Confidence intervals		K-W test	*Post hoc* analysis		
								*p* value	K + VS. K=	K + VS. K−	K = VS. K−
Walk											
Vertical impulse (VI) [Newton-second]	VI _LR	K+	3468.59	358.9	3485.94	2765.14	4172.04	0.009	0.891	0.009	0.006
		K=	3523	272.28	3454.71	2989.33	4056.68				
		K−	3419.51	266.21	3424.18	2897.74	3941.29				
Average max force (AVGF) [%body weight]	AVGF_LR	K+	41.67	4.51	41.59	32.84	50.51	0.048	0.902	0.038	0.028
		K=	41.75	4.34	41.19	33.25	50.25				
		K−	40.66	4.98	39.68	30.89	50.43				
Average max force (AVGF) [%body weight]	AVGF_RR	K+	40.01	4.52	39.62	31.16	48.86	0.042	0.487	0.078	0.014
		K=	40.4	4.25	40.18	32.07	48.74				
		K−	39.23	4.56	38.21	30.3	48.17				
Trot											
Step width (SWD) [cm]	SWD_Hind	K+	5.17	1.31	4.98	2.6	7.75	0.008	0.008	0.942	0.006
		K=	4.67	1.63	4.53	1.48	7.86				
		K−	5.21	1.44	5.06	2.39	8.04				
Hind reach (Hind R) [cm]	Hind R_L	K+	5.4	20.64	−0.74	−35.05	45.86	0.02	0.005	0.277	0.089
		K=	0.33	14.08	−3.39	−27.27	27.92				
		K−	2.51	14.29	−2.12	−25.5	30.53				
		Symmetry index (SI) [%]	SI _Front	K+	4.32	4.11	2.97					−3.73	12.37	0.022	0.012	0.821	0.023
K=	5.1			3.5	4.88	−1.76	11.96										
K−	4.17			3.51	3.23	−2.71	11.04										

Three different taping methods: facilitate taping period K+, no effect taping period K=, inhibit taping period K−; LR, left rear, RR, right rear; L, left; SD, Standard deviation; Green background means kinetic related; Yellow background means temporospatial related; K-W test, Kruskal–Wallis test.

There was no significant difference in the velocity, which is expected due to the group comparison of dogs of varying sizes. Similarly, no differences were observed in stride length and step length due to the cross-over group comparison over time. There were no differences in stance and swing phase during both walk and trot with different taping methods.

Regarding the comparison between taping with hair (H) and with clipped (C), one dog experienced an idiopathic seizure during the hair growth period. The owner was unwilling to return for the taping with hair session, and the dog’s clinical condition was not stable enough to continue in the study. Seven dogs completed the study. The comparison of 29 variables in the walking trials revealed 9 significant differences (*p* < 0.05) between haired and clipped taping conditions (*n* = 7). These differences were observed in vertical impulse of the left hind limb (C > H), stride length (C > H), step length of the left hind limb (C < H) and right hind limb (C < H), step width of the hind limb (C > H), hind reach of the left (C < H) and right limbs (C < H), stance phase of the left hind limb (C > H), and swing phase of the left hind limb (C < H). In the trotting trials (*n* = 7), two significant differences (*p* < 0.05) were found: vertical impulse of the right front limb (C > H) and step width of the hind limb (C > H). There were two significant differences (*p* < 0.05), which were on the vertical impulse of the right front (C > H) and step width of the hind (C > H) on the measurements between with hair and clipped taping on trot (*n* = 7). (Table 3; for detailed data, see Supplementary Material).

**Table 3:** The comparison of the measurements showing significant difference with clipped/haired facilitate taping (*n* = 7).

Clipped				Haired	
		Mean	SD	Median	Confidence intervals		Mean	SD	Median	Confidence intervals		*p*-value
In walk												
Vertical impulse (VI) [newton-second]	VI _LR	3456.28	456.29	3475.54	2559.66	4352.9	3364.93	451.31	3366.71	2478.09	4251.76	0.03
Stride length [cm]	Stride_length	65.49	15.89	70.78	34.26	96.73	67.25	15.1	73.8	37.58	96.93	0.02
Step length (SL) [cm]	SL_LR	32.47	8.4	35.15	15.95	48.98	33.59	7.58	36.42	18.7	48.49	0.04	SL_RR	32.62	7.9	34.51	17.1	48.14	34.36	6.48	36.42	21.63	47.09	0.01
	Step width (SWD)[cm]	SWD_Hind	6.33	1.73	6.49	2.94	9.73	5.74	1.74	5.67	2.33	9.15	0.04
Hind reach (Hind R) [cm]	Hind R_L	11.53	6.03	12.42	−0.32	23.38	14.06	6	13.97	2.26	25.85	0.05
	Hind R_R	10.98	6.44	13.41	−1.67	23.63	12.58	7.28	13.55	−1.73	26.88	0.01
Stance phase (ST) [%]	ST _LR	63.31	2.36	63.6	58.67	67.96	60.73	6.4	61.73	48.15	73.31	0.01
Swing phase (SW) [%]	SW _LR	36.69	2.36	36.4	32.04	41.33	39.27	6.4	38.27	26.69	51.85	0.01
In trot												
Vertical impulse (VI) [newton-second]	VI _RF	6286.43	1622.55	5972.28	3098.12	9474.74	5726.39	921.24	5807.85	3916.14	7536.63	0.01
Step width (SWD) [cm]	SWD_Hind	5.38	1.47	5.19	2.49	8.27	5.08	1.38	5.36	2.37	7.79	0.04

LR, left rear; RR, right rear; RF, right front; L, left; SD, Standard deviation; Green background means kinetic-related; Yellow background means temporospatial-related.

**Figure 3:**
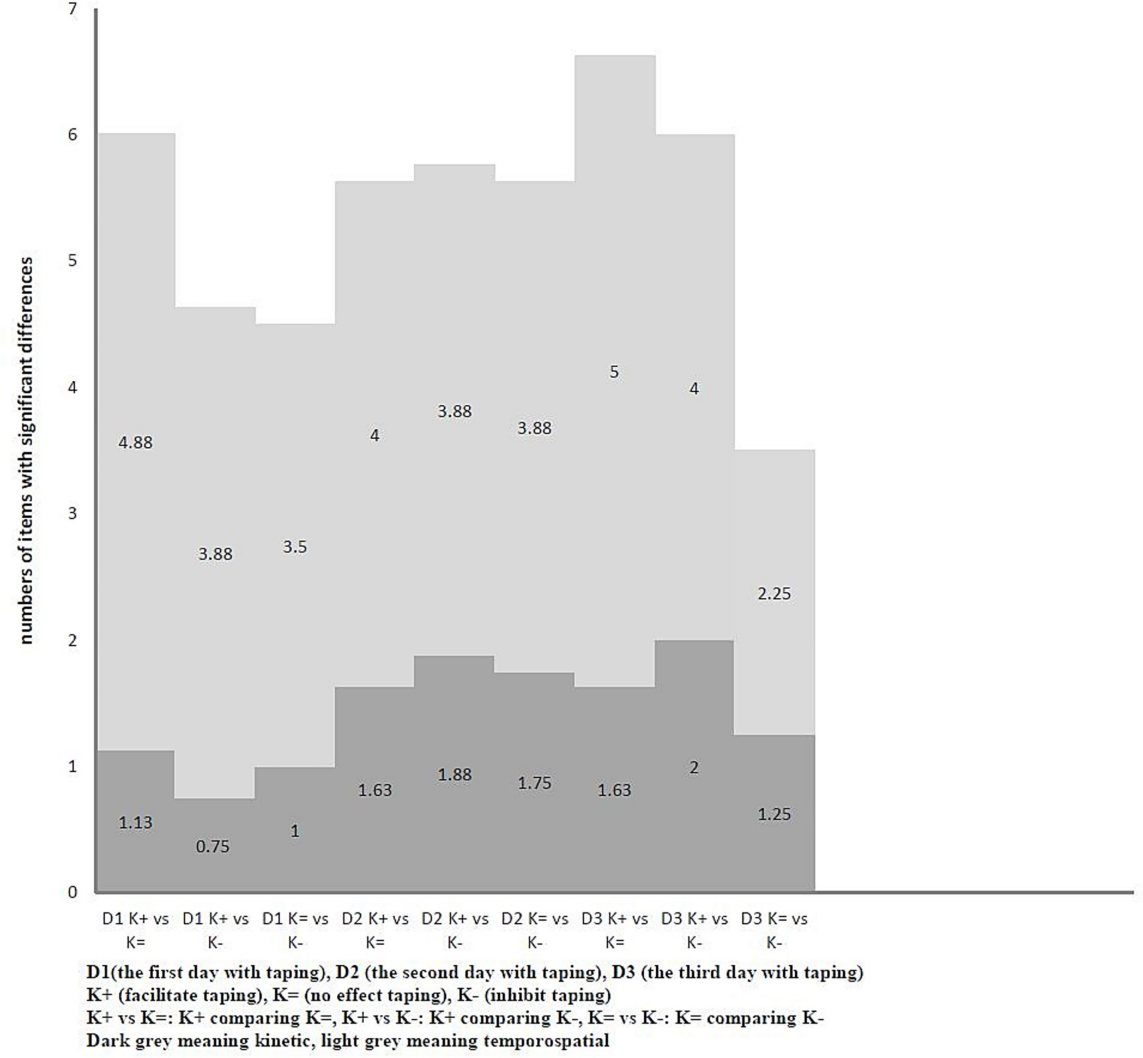
The average number of items with significant differences compared to different taping methods with each other on the same day under walk (*n* = 8).

**Figure 4:**
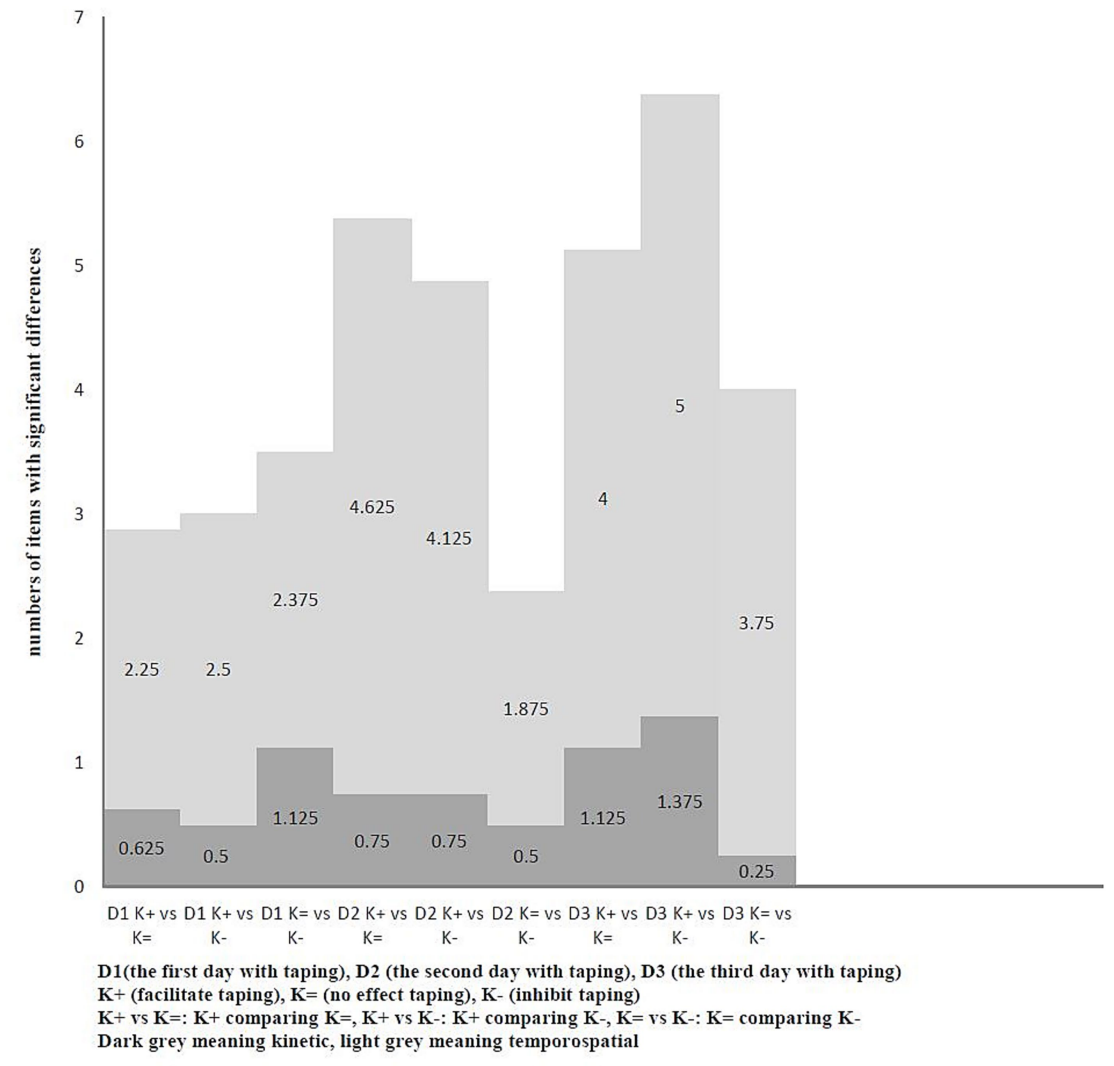
The average number of items with significant differences compared to different taping methods with each other on the same day under trot (*n* = 8).

Comparison of taping methods on the same day among all dogs (*n* = 8) showed that the average number of significantly different items during walking ranged from 4.5 to 6.01 on Day 1. The greatest differences were observed between K + and K=, followed by K + versus K−, with K = versus K − showing the fewest differences. On Day 2, the range was 5.63 to 5.76, with K + versus K − showing the highest differences, followed by K + versus K=, and K = versus K − being the lowest. On Day 3, the range was 3.5 to 6.63, again with K + vs. K = being the highest, followed by K + versus K−, and K = vs. K − being the lowest. When trotting, the number of items with significant differences (*p* < 0.05) across taping methods on Day 1 ranged from 2.875 to 3.5, with K = versus K − showing the most differences, followed by K + versus K−, and K + versus K = showing the fewest. On Day 2, the range was 2.375 to 5.375, with K + versus K = highest, followed by K + versus K−, and K = versus K − was the lowest. On Day 3, it ranged from 4 to 6.375, with K + versus K − highest, followed by K + versus K=, and K = versus K − was the lowest. The patterns of these comparisons with different taping methods were inconsistent. Likewise, the distributions of kinetic and temporospatial variables also varied inconsistently. (Figures 3, 4; for detailed data, see Supplementary Material).

## 4 Discussion

Kinesiology taping (KT) is widely used in human sports medicine due to its perceived benefits in injury prevention and muscle performance enhancement. This concept has recently been applied to healthy dogs. Two key questions remain: (1) Does taping direction (facilitation vs. inhibition) affect dogs similarly to humans? (2) Is KT effective when applied over fur? While KT use is growing, scientific evidence in dogs is limited.

A study by Noel et al. was among the first to investigate KT in healthy canines. They applied tape with 25% stretch in a proximal-to-distal direction across the tarsal joint, aligning with a facilitation technique, to assess its impact on pelvic limb movement. Gait analysis performed within 2 h of application revealed no significant changes in kinetic or kinematic parameters during walking, trotting, or stepping over obstacles (34). Unlike their study, this research evaluated the extended effects of different KT applications on canine gait, incorporating both kinetic and temporospatial measurements over multiple days.

To our knowledge, this is the first study to investigate the sustained effects of different KT methods in healthy dogs over an extended period. The CODI results showed that the taping did not affect observable mobility in healthy dogs. Given the subjective nature of CODI, static and dynamic objective analyses (stance analyzer and pressure-sensing walkway) were also performed (35). These objective analyses showed no significant differences in stance based on taping method in the taped leg. This aligns with human research where KT’s immediate and delayed effects on femoral quadriceps performance, balance, and lower limb function in healthy women found no change in static balance (36). While another study in humans demonstrated that KT benefits dynamic activities in children with cerebral palsy, it had no effect on static activities (37). The taping did not affect the static stance results regardless of the health status in human studies. Interestingly, we observed effects on non-taped legs (*p* < 0.05), suggesting that dogs perceive the sensation of the tape while standing and adjust their other legs to maintain balance. Unlike humans, who can verbalize these sensations, dogs’ responses are reflected in stance changes, likely representing anticipatory postural adjustments where the central nervous system reorganizes the activity of individual postural muscles to compensate, ensuring stance balance (38).

It has been proposed that KT may facilitate or inhibit muscle function depending on the taping direction. However, study findings are inconsistent; many report no significant facilitatory or inhibitory effects of KT on muscle strength or electromyographic activity (39–42). In our study, the PSW analysis, our dynamic measurement showed that taping with the inhibition technique (insertion to origin) produced the weakest effects on kinetic variables, irrespective of whether the dog was walking or trotting. While our study showed minimal overall effects, different taping methods may still influence gait patterns differently in dogs. Therefore, applying tape in the incorrect direction is unlikely to yield beneficial outcomes.

When comparing different taping methods on the same day during walking and trotting, the variable changes were not consistently associated with specific days or taping types. The specific gait variables, whether kinetic or temporospatial, showed that significant differences varied independently. We assume that the dogs likely adjusted their neuromuscular activity and limb locomotion in response to the subtle stimulation from the thin elastic tape, helping maintain their gait stability in a healthy state. These findings indicate that taping effects varied across days when the tape remained in place for 48 h.

This prompts a question that warrants further investigation: when taping is needed for several consecutive days, is it more effective to reapply the tape daily or to leave it on to preserve consistent outcomes? A human study by Sheikhi et al. (43) investigated the immediate effects of various taping tensions (0, 50, and 75%) on tuck jump performance in 75 active individuals. The tape was applied bilaterally to lower limb muscles, such as the gastrocnemius, biceps femoris, vastus lateralis, vastus medialis, rectus femoris, and gluteus medius, for 72 h. No significant differences were observed between the KT groups at the immediate, 24-h, and 72-h time points (43). In contrast, our study found that different taping methods in dogs affected gait patterns variably across days during both walking and trotting, although the overall taping effects remained limited.

Quadrupedal dogs, unlike bipedal humans, possess a greater compensatory ability. A study of 39 amputee dogs using pressure-sensitive walkways examined forelimb and hindlimb amputations at different levels. High amputations involved hip or scapular disarticulation, while low amputations were distal limb removals. The level of forelimb amputation did not affect weight distribution, but high hindlimb amputations led to increased loading on the opposite limb (30). Dogs with permanent limb loss compensate consistently. In contrast, our study showed that healthy dogs responded inconsistently to temporary unilateral taping on the biceps femoris.

The effect of KT on the enhancement of muscle activity has been observed in healthy human athletes experiencing muscle soreness. Kirmizigil et al. (44) published a study investigating the effects of KT on delayed onset muscle soreness after exercise. The tape was applied bilaterally to the rectus femoris of healthy amateur athletes. The results indicated some beneficial effects on performance and balance (44). For this reason, KT is commonly used by competitive athletes to alleviate soreness and maintain performance, rather than to increase strength.

In humans with musculoskeletal diseases, there is evidence suggesting that kinesiology tape may positively affect muscle strength during weight-bearing movements (45). During non-weight-bearing movements, a human study by Cho et al. (46) demonstrated that KT therapy applied to patients with chronic lateral epicondylitis resulted in improved pain-free grip strength. The impact of KT on strength evaluation seems to differ between humans and animals (46). In humans, strength is typically assessed using open-chain exercises, whereas in animals, it is usually evaluated during closed-chain activities such as walking or trotting.

Vithoulka et al. (42) showed that the application of KT to the anterior surface of the thigh in the direction of vastus medialis, lateralis, and rectus femoris fascia could increase eccentric muscle strength. However, a study in horses investigating the effects of KT on the trajectory of the forelimb and the muscle activity of the *M. brachiocephalicus* and the *M. extensor carpi radialis* at the walk and trot found no significant differences among no tape, with tape, and post tape conditions (47). To date, no research has demonstrated an enhancement of muscle activity in healthy dogs using KT. In our study, healthy dogs performed activities with taping at the walk and trot, but no consistently significant results were obtained. The healthy dogs appear to maintain homeostasis as effectively as possible.

Our results showed that some kinetic and temporospatial variables had significant differences (*p* < 0.05) when comparing three different taping methods across different days. However, the variable items changed inconsistently. This could indicate that intrinsic body modulations were caused by the stimulation of the skin via taping. This inner drive of movement generated by the elastic tape might be related to Ia afferent neurons and alpha motor neurons (48) and the related reflex. Bagheri et al. (49) examined the H-reflex recruitment curve of the gastrosoleus muscle under several conditions: no treatment (control), KT, KT applied over skin with topical anesthesia, topical anesthesia alone, and sham taping without tension. Their results showed that KT, whether applied to normal or anesthetized skin, enhanced the H-reflex parameters. In contrast, topical anesthesia alone suppressed these parameters, while sham taping had no effect. These findings indicate that kinesiology taping promotes muscle activity, likely by stimulating cutaneous receptors that influence the motor neuron pool of the gastrosoleus muscle (49). Further research is needed to explore its influence on sensory input and motor responses in canine models.

The comparison between taping with hair and clipped is interesting. In our analysis, the results suggest that the presence or absence of hair influences the effect of taping. To explore how taping over hair might still be effective, we focused on kinetic parameters such as vertical impulse and peak vertical force on the applied limb, which directly reflect changes in force. Gillette and associates (50) mentioned that both peak vertical force and vertical impulse are indicators of limb usage. In our study, we only assessed the effects of facilitative taping on the left hind limb during both walk and trot, comparing conditions with hair and clipped. Since a dog’s fur is considered a physical barrier, it may diminish the effectiveness of taping. This decision is supported by a previous study conducted on healthy young adults, which compared three groups: one receiving kinesiology tape from origin to insertion (facilitation), another from insertion to origin (inhibition), and a control group with no taping. The results showed no changes in the inhibition or control groups, while facilitation taping led to increased muscle tone, elasticity, and stiffness (40). Our findings demonstrated a significant decrease (*p* < 0.05) in force-related variables in walk and trot when hair was present, indicating a diminished or minimal taping effect. Other variables indirectly related to the taped limb exhibited variable changes, potentially indicating compensatory adaptations. Overall, the results suggest that hair reduces the efficacy of kinesiology taping and that application on clipped areas is necessary to achieve the intended therapeutic effects.

Theoretically, KT offers contraction on the skin mimicking a second layer of skin, which produces tactile input. It is similar to the innocuous mechanical stimulation of low-threshold mechanoreceptors by which skin or hair react to perceive the stimuli—this is as opposed to the high-threshold mechanoreceptors that respond to harmful mechanical stimulation (51). In hairy skin, tactile stimuli are transduced through three types of hair follicles (51). It has been suggested that KT may stimulate hair follicles to trigger signal input in dogs, as noted by the Kinesio Group (52). In our study, notable differences were found between taping on shaved versus unshaved skin, even though overall mobility was unaffected. It is still unclear whether the direct skin contact with the tape does not arouse enough stimulation, or if the presence of hair itself interferes with the effectiveness of kinesiology taping—both possibilities need to be explored further. Additionally, the exact influence of hair follicle stimulation on how dogs respond to KT remains undefined and calls for more research.

Our results offered partial support for the initial hypothesis. As the first study to examine various kinesiology taping (KT) techniques in healthy dogs over time, it identified only subtle and inconsistent impacts on gait kinetics and temporospatial parameters, indicating that taping has a limited effect on canine movement. Interestingly, non-taped limbs showed compensatory changes, likely due to proprioceptive feedback. The inconsistent variable changes across different taping methods in healthy dogs did not align with the expected facilitation or inhibition mechanisms. Furthermore, the presence of hair significantly affected taping effectiveness, underscoring the importance of skin stimulation in KT outcomes. These results emphasize the adaptive capacity of healthy dogs and point to the need for additional studies in clinical settings.

### 4.1 Limitations and conclusion

There were notable limitations to our study. The main limitation of this study was the small number of dogs, which reduced the statistical power. Recruiting a large sample for a prospective study requiring multiple three consecutive days of visits was challenging, as most owners could not commit to the protocol due to time constraints.

Another limitation was the diverse range of breeds and sizes, resulting in a heterogeneous sample population. The KT area was applied in proportion to the target muscle by length. However, no research exists that determines the ideal KT area in relation to dog size or taping area. The same tape width was used across different-sized dogs, and the adequacy of this dosage remains uncertain. In addition, size and breed differences, hair length also posed a challenge in assessing KT on fur. This study included two short-coated dogs, two combination-coated dogs, three double-coated dogs, and one silky-coated dog, demonstrating diversity.

Another limitation of this study is the difficulty in evaluating muscle strength or activity in dogs. KT research in humans has focused on several outcomes, including muscle activity, neuromuscular efficiency, rate of force development, and movement performance (53). Among these, movement performance—such as ground reaction force—is the most feasible and convenient to assess in dogs. At present, gait analysis remains the main approach for evaluation; however, it does not fully reflect the functional performance of the biceps femoris, a key muscle involved in stifle extension. Wireless electromyography (EMG) offers a dependable means of assessing individual muscle activity and can objectively detect kinematic changes influenced by specific muscles. Nevertheless, since KT functions through tactile stimulation of the skin to activate nerves, applying any device to the skin under dynamic conditions, such as EMG sensors, may interfere with the taping effect, presenting a dilemma.

Another limitation was that the PSW sensors measured the foot at a frequency of 100 Hz, which only recognizes walking and trotting, not running. We are unsure if the KT effect differs during high-speed motion, given the potential compensation of limbs at higher velocities when running.

We concluded that in healthy dogs, KT had no impact on overall mobility or stance of the affected leg, with only minor effects observed during walking and trotting. Different KT application methods led to slight, inconsistent changes in gait pattern. Variability in kinetic and temporospatial data was expected due to the adaptability of healthy dogs. These fluctuations likely reflect natural gait variability. While KT may influence nerve input and trigger compensatory limb adjustments, its overall impact on performance appears limited. Interestingly, only a decreased vertical impulse on the taped limb when taping with hair was observed, warranting further research on KT’s effect on hair follicles.

### 4.2 Future work

The results in this study lay an essential foundation for future studies examining other muscle groups that significantly contribute to muscle atrophy and functional decline in dogs. Consideration should be given to applying double or triple taping on counteracting muscle sides that work in synchrony during extension or flexion. Several parameters, both kinetic and kinematic, showed significant variability. The relationship between these factors and the dog’s response to KT needs further exploration. It remains unclear how these elements influence performance. To establish conclusive results, a more comprehensive study design is required. Future assessments could include evaluating the effects of KT on fur, potentially by applying tape to smooth-coated dogs like French Bulldogs or Pointers to study its impact on hair follicles.

## Data Availability

The original contributions presented in the study are included in the article/Supplementary material, further inquiries can be directed to the corresponding author.
